# Rapid and Efficient Generation of Transgene-Free iPSC from a Small Volume of Cryopreserved Blood

**DOI:** 10.1007/s12015-015-9586-8

**Published:** 2015-05-08

**Authors:** Hongyan Zhou, Hector Martinez, Bruce Sun, Aiqun Li, Matthew Zimmer, Nicholas Katsanis, Erica E. Davis, Joanne Kurtzberg, Scott Lipnick, Scott Noggle, Mahendra Rao, Stephen Chang

**Affiliations:** 1 The New York Stem Cell Foundation Research Institute, New York, NY 10032 USA; 2 Center for Human Disease Modeling, Division of Pediatric Blood and Marrow Transplantation, Durham, NC 27710 USA; 3 Department of Pediatrics, Division of Pediatric Blood and Marrow Transplantation, Durham, NC 27710 USA

**Keywords:** Reprogramming, Peripheral blood, Cord blood, Sendai viral vector, Genomic rearrangement, GMP, Cell therapy, iPSC

## Abstract

Human peripheral blood and umbilical cord blood represent attractive sources of cells for reprogramming to induced pluripotent stem cells (iPSCs). However, to date, most of the blood-derived iPSCs were generated using either integrating methods or starting from T-lymphocytes that have genomic rearrangements thus bearing uncertain consequences when using iPSC-derived lineages for disease modeling and cell therapies. Recently, both peripheral blood and cord blood cells have been reprogrammed into transgene-free iPSC using the Sendai viral vector. Here we demonstrate that peripheral blood can be utilized for medium-throughput iPSC production without the need to maintain cell culture prior to reprogramming induction. Cell reprogramming can also be accomplished with as little as 3000 previously cryopreserved cord blood cells under feeder-free and chemically defined Xeno-free conditions that are compliant with standard Good Manufacturing Practice (GMP) regulations. The first iPSC colonies appear 2–3 weeks faster in comparison to previous reports. Notably, these peripheral blood- and cord blood-derived iPSCs are free of detectable immunoglobulin heavy chain (IGH) and T cell receptor (TCR) gene rearrangements, suggesting they did not originate from B- or T- lymphoid cells. The iPSCs are pluripotent as evaluated by the scorecard assay and in vitro multi lineage functional cell differentiation. Our data show that small volumes of cryopreserved peripheral blood or cord blood cells can be reprogrammed efficiently at a convenient, cost effective and scalable way. In summary, our method expands the reprogramming potential of limited or archived samples either stored at blood banks or obtained from pediatric populations that cannot easily provide large quantities of peripheral blood or a skin biopsy.

**Electronic supplementary material:**

The online version of this article (doi:10.1007/s12015-015-9586-8) contains supplementary material, which is available to authorized users.

## Introduction

The emergence of iPSC technology, with the capability of iPSC to undergo unlimited self-renewal and differentiation into any type of cell, has great potential to advance translational applications including stem cell therapies and generation of large-scale collections of lines for research. The availability of precisely generated iPSC-derived functional cells to replace or repair damaged tissues/organs will likely affect hematopoietic diseases therapies, facilitate the treatment of neurologic disorders, cardiovascular, liver, and retinal diseases, and possibly diabetes [1–4]. In fact, the first clinical study of cell-based therapy using iPSC derived from patients to treat blindness started in September 2014 in Japan (http://www.nature.com/news/japanese-woman-is-first-recipient-of-next-generation-stem-cells-1.15915). This program uses autologous iPSC-derived retinal pigment epithelium for therapy. While this mechanism theoretically alleviates many immunological issues owing to the fact that it is patient-specific, it requires significant time and is very expensive, thereby making it impractical for many conditions, especially for people with acute disease conditions. An alternative strategy is haplobanking of allogeneic iPSC lines that could partially match a majority of potential patients with limited immune suppression [5, 6]. However, finding donors that have human leukocyte antigen (HLA) homozygosis is challenging unless one has access to donor registries of pretested individuals/samples. The availability of a broad type of HLA-typed cells in peripheral blood and cord blood banks suggest that these sources are ideal for such an effort [5, 6]. Since blood units stored in banks are mainly for clinical use, it is important to utilize a small portion of the sample and not compromise the ultimate therapeutic use of the blood unit.

The identity of the cell of origin from blood that undergoes transformation into an iPSC could also be important. White blood cells in peripheral blood, the nucleated cells that can be reprogrammed, contain a mixture of hematopoietic stem cells (CD133+ and 34+), T cells (CD3+), B cells (CD19+), T helper cells (CD4+), macrophages (CD14+), erythroblasts (CD71+), and myeloid progenitor cells (CD13+). As an attractive candidate that contains an intact genome for iPSC generation, CD34+ blood progenitors have been reprogrammed into pluripotent cells by several labs using different approaches [7–10]. However, the use of cytokines like G-CSF mobilization is often required to get a reasonable number of CD34+ cells into the peripheral circulation for collection, and stored samples may have an inadequate concentration when small volumes are used. Thus the availability of source material limits the broad application of using CD34+ cells to generate iPSCs. T-lymphocytes are another broadly used blood-derived cell source for reprogramming, even though cells need to be activated before reprogramming induction and often have much lower reprogramming efficiency. However, just like B-lymphocytes, T-lymphocytes harbor a set of gene rearrangements that may result in skewed development of T cells and perhaps other lineages [11], and even worse, could develop T cell lymphomas spontaneously [12]. Alternatively, expanded erythroblasts are a promising source for reprogramming using lentivirus, Sendai virus, or Epsterin-Barr virus (EBV)-based vector approaches [13–15]. However, the prolonged cell expansion and selection with an erythropoiesis-stimulating environment before the reprogramming step significantly increases the cost [16]. In addition, the use of unstandardized reagents and the involvement of feeders for iPSC generation will make it challenging to comply with good manufacturing practice (GMP) regulations for clinical grade iPSC production.

In this study, we aimed to generate gene-rearrangement-free iPSC clones from a small volume of cryopreserved blood cells using a gene-integration free method with GMP grade reagents. To do this, we compared three blood cell storage methods for their cell recovery rate and cell viability, and then carried out a screen of cell subsets that form during peripheral blood mononuclear cell (PBMC) expansion using a collection of cell surface markers that are expressed by specific blood lineage cells. Antibody mediated identification of cell subsets allowed us to characterize separate defined subpopulations. Two types of gene-rearrangement-free blood cells, CD13+ and CD71+, were successfully and efficiently reprogrammed. Enriched and cloned blood-derived iPSCs were then tested for their pluripotent potential. Gene expression analysis and functional assay results did not show any substantial difference among those iPSC lines when compared to reference embryonic stem cell (ESC) lines. Moreover, the described method was adapted to a 96-well plate format, allowing for medium/high-throughout iPSC derivation for scientific purposes and potentially for cell therapies.

## Material and Methods

### Blood Cell Preparation and Storage

All human whole blood samples were obtained from New York Blood Center, cord blood samples were obtained from Carolinas Cord Blood Bank, with approval of the local research ethics committee.

For blood cell storage test, samples of peripheral blood were processed by three methods: 1) One volume of peripheral blood samples was mixed with one volume of Synth-a-freeze (SAF, Life Technologies), the mixture was centrifuged at 600 *g* for 5 min to get the cell pellet. The pellet was resuspended with SAF and stored in the liquid nitrogen tank for future application; 2) peripheral whole blood samples were added to 10 % DMSO (Sigma) and stored in the liquid nitrogen tank; 3), peripheral blood samples were processed using the standard, 8 mL Vacutainer Cell Processing Tubes (BD Biosciences) according to the manufacturer’s protocol. Briefly, the PBMC-containing upper phase was collected and washed with PBS, centrifuged at 600 *g* for 15 min. The cell pellets were resuspended with SAF, and stored in the liquid nitrogen tank. For the peripheral blood cells used for cell fate characterization and reprogramming experiments, PBMCs were prepared by method 3. For the cord blood samples used for reprogramming experiments, cord blood was collected from a segment attached to the cord unit using syringe and needle. A total of about 20 μl cord blood samples were lysed in 1 ml of 1 × red blood cell lysis buffer (eBioscience) for 10 min before centrifuging at 600 *g* for 5 min. The lysis buffer was removed after centrifugation. The cell pellets were resuspended with cell expansion medium and seeded into low attachment plates.

### Blood Cell Reprogramming

**Fig. 1:**
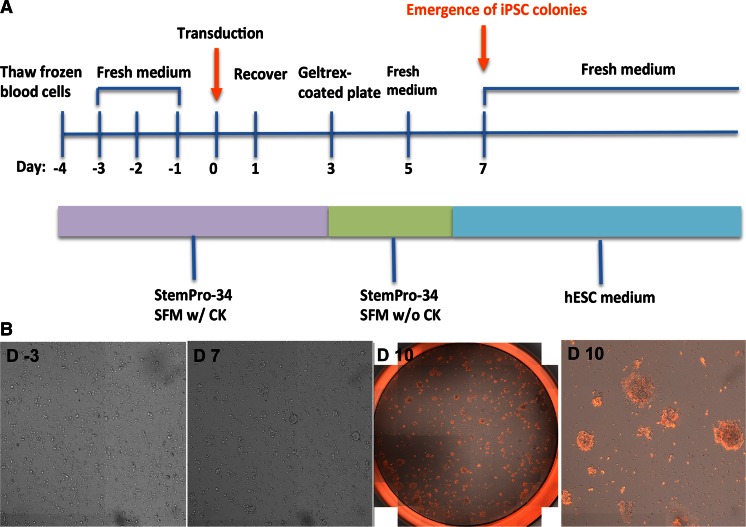
Derivation of human iPSCs from human peripheral blood samples. **a** Scheme for reprogramming human peripheral blood. **b** Live whole-well (24-well plates) and zoomed representative images of peripheral blood at different stages during reprogramming process. Images were taken by Celigo software. **D-3:** Three days before transduction; **D7:** Seven days after transduction; **D 10:** Ten days after transduction

Blood cell expansion medium contained StemPro-34 SFM (Life Technologies) supplemented with 100 ng/ml stem cell factor (SCF, R&D Systems), 100 ng/ml FLT3 (eBiosciences), 20 ng/ml interleukin-3 (IL3, Cell Signaling), and 20 ng/ml interleukin-6 (IL6, Cell Signaling). Medium was changed every day for 4 days (Day -4 to Day -1, Fig. 1a) by centrifugation to remove the medium and replacing with fresh medium. After 4 days cell expansion (Day 0), cells were transduced by *Oct4, Sox2, Klf4*, and *c-Myc* Sendai viral vectors (CytoTune-iPSC 2.0 Sendai Reprogramming Kit, Life Technologies) at a multiplicity of infection (MOI) of 5. The transduction was performed in StemPro-34 SFM supplemented with cytokines containing 4 μg/mL of Polybrene by centrifugation at 2000 RPM for 30 min. The day after transfection (Day 1), Sendai Viruses were removed by centrifuging the cell suspension. The cells were resuspended with fresh StemPro-34 SFM supplemented with cytokines for 2 days. The next day (Day 3), the cells were then collected by centrifugation, resuspended with StemPro-34 SFM without cytokines, and seeded onto Geltrex-coated plates at the targeted densities. The medium was refreshed every other day. From Day 6–7, the medium was changed to customized human ESC medium Freedom-1 (Life Technologies) with daily medium changes. Once the ESC like TRA-1-60+ iPSC emerged, the colonies were manually picked and replated onto Geltrex-coated plates for expansion. For extremely small number of cord blood cell reprogramming (e.g., 3000 cells), cells were reprogrammed using the same method as above except using a higher MOI of 15.

### iPSC Purification by CD13+, CD71+ Cell Depletion

A negative selection procedure was chosen for purification of blood-derived iPSC. Single cell suspensions of whole reprogramming events were first labeled with CD71 MicroBeads (Miltenyi, #130-046-201) to allow AutoMACS depletion of CD71+ cells. The enriched cells were next labeled with biotinylated monoclonal antibody CD13 (Miltenyi, #130-103-768), followed by incubation with anti-biotin microbeads (Miltenyi) according to manufacturer’s recommendations and depleted using an AutoMACS (Miltenyi). The cell purity was confirmed by flow cytometry.

### Flow Cytometric Analysis

Cultured cells were dissociated with 0.5 mM EDTA (Life Technologies). Single cell suspensions were collected by centrifugation and suspended at 3 × 10^4^ to 1 × 10^5^ in 100 μL of cold PBS containing 2 mM EDTA and 0.5 % BSA (FACS buffer). Specifically labeled antibodies or appropriate isotype controls were added, and cells were incubated further for 30 min. Cells were washed once and resuspended in 200 μL of FACS buffer. To determine pluripotency of derived iPSCs, cells were stained with CD13 (BD Biosciences, #555394; 1:100 dilution), SSEA-4 (BD Bioscience, #560219; 1:100 dilution), TRA 1-60 (BD Bioscience, #560173; 1:100 dilution) and DAPI (Life Technologies; 1μg/mL). To determine the cell fate before and after blood cell reprogramming, cell were labeled with PE-conjugated, FITC-conjugated, APC-conjugated or V450 –conjugated antibodies against CD3 (BD Biosciences #555333), CD4 (BD Biosciences #553730), CD13 (BD Biosciences #340686), CD13 (BD Biosciences #558744), CD14 (BD Biosciences #553740), CD15 (BD Biosciences #561584), CD19 (BD Biosciences #553785), CD34 (Miltenyi Biotec #130-081-002), CD45 (BD Biosciences #560368), CD71 (BD Biosciences #551374), TRA-1-60 (BD Biosciences #560380, #563187), and SSEA-4 (BD Bioscience, #560219, #560308). Stained cells were analyzed on a 5 laser BD Biosciences ARIA-IIu™ SOU Cell Sorter. The resulting data were analyzed using FlowJo software (Treestar).

### Embryoid Body Formation

To form embryoid bodies (EBs), undifferentiated hiPSC were dissociated with 0.5 mM EDTA (Life Technologies) and were collected and resuspended in Freedom-1 medium supplemented with 1 μM Thiszovivin (Stemgent). The cells were added to low attachment 24-well plates and incubated for 24 h at 37 °C in a 5 % CO_2_ atmosphere to form EBs. The medium was change to hEB medium consisting of 80 % DMEM/F12 medium, 20 % knockout serum replacement (KSR, Invitrogen), 1 % non-essential amino acids (NEAA, Invitrogen), 1 mM GlutaMAX (Invitrogen), 0.1 mM β-mercaptoethanol (Sigma) and refreshed every day. After 16 days of incubation, total RNA was isolated from EBs. The expression levels of each differentiation and pluripotent markers were determined using Nanostring, as described below.

### Gene Expression Analysis of iPSCs

Nanostring (Nanostring Technologies) and Scorecard analysis was performed as described [17]. iPSCs were cultured in Freedom-1 medium before RNA isolation. To measure their differentiation propensities, undifferentiated blood-derived iPSC and EBs were collected and total RNA was extracted using RNeasy plus kit (Qiagen). One hundred nanograms of RNA was profiled on the Nanostring nCounter system (Nanostring technologies) according to the manufacturer’s instructions. A custom nCounter codeset for Pluri25 covering 25 genes that evaluate cell pluripotency and a custom nCounter codeset for 3GL covering 83 probes that monitor differentiation were used. Lines were analyzed in biological duplicates for each hESC reference line and iPSC line. All data were normalized analyzed with nSolver Analysis Software (Nanostring Technologies, USA).

### Immunohistochemistry Staining

Cell lines were rinsed with 1X PBS, fixed with 4 % paraformaldehyde (Santa Cruz, #sc-281692) in PBS for 5 min at room temperature. Nonspecific binding sites were blocked by incubation with PBST (PBS supplemented with 0.1 % Triton X-100) containing 10 % donkey serum (Jackson Labs, #017-000-1210) for 30 min at room temperature. Cells were subsequently incubated overnight at 4 °C in PBST containing 10 % donkey serum and specific primary antibodies: 1:500 anti-Human OCT4 (Stemgent, #09-0023), 1:100 anti-human NANOG (Cell Signaling Technologies, #4903), 1:250 anti-human SSEA4 (Abcam, #ab16287), 1:500 anti-TRA-1-60 (Life Technologies), 1:200 anti cTnT, 1:200 anti-ACTIN, 1:200 anti-SOX1, 1:200 anti-SOX2, 1:200 anti-NESTIN, 8 ng/ml anti-SOX17, 10 ng/ml anti-ALB, 1:500 anti-Sendai Vector (MBL, #PD029). Following a 3 times wash with PBS, cells were incubated with one of the following secondary antibodies: Alexa Fluor® 488 donkey anti-Mouse (#A-21202; 1:1000 dilution), Alexa Fluor® 555 donkey anti-goat IgG and Alexa Fluor® 555 donkey anti-rabbit IgG (#A-21428; 1:1000 dilution). After washing 3 times with PBS, the samples were incubated for 10 min with Hoechst (1 μg/ml) in PBS, followed by a final wash in PBS. Fluorescence images were captured with the Celigo, Nikon Eclipse TE 2000-U or Olympus BX41 fluorescent microscopes.

### DNA Isolation

DNA isolation from the cell pellets was achieved using the High Pure Template PCR Template Preparation Kit (Roche, #11796828001) per manufacturer’s instructions with the following modifications: 1) cells were treated with 4 μL of RNase (Qiagen, #19101) for 2 min while resuspended in PBS; 2) DNA was eluted in 30 μL of water.

### T-Cell and B-Cell Rearrangement Assay

PCR was used to identify clonal TCR and IG gene rearrangements using a commercially available method employing multiple multiplexed PCR tubes that was originally developed as the result of a large European BIOMED-2 collaborative study (Invivoscribe Technologies). For IGH gene detection, PCR (35 cycles of 94 °C for 30 s, 55 °C for 30 s, and 72 °C for 1 min) was performed using 1 U of AmpliTag Gold polymerase (Life Etchnologies) in a 27.5 μl reaction with 100 ng of genomic DNA and 25 μl of primer master mix. For TCRB, TCRD, and TCRG genes detection, PCR (35 cycles of 94 °C for 45 s, 60 °C for 45 s, and 72 °C for 90 s) was performed. All PCR started with initial denaturation at 95 °C for 7 min and ended with final extension at 72 °C for 10 min before holding at 4 °C. PCR products were detected on 2 % agarose gels.

### In Vitro Directed Differentiation Assay

The derived cells were induced to differentiation into NSC [18, 19], cardiomyocyte [20], as well as hepatocyte-like cell [21] as described below.

Directed NSC differentiation procedure consists of a 11-day neural induction, as described previously [18, 19]. iPS cells were seeded at a density of 1.5 × 10^6^ per well of 6-well plates when initializing the neural differentiation. The cultures were then treated with dual-SMAD inhibitors, LDN193189 (100 nM) and SB421542 (10 μM), sonic hedgehog (SHH, 200 ng/mL), puromorphamine (2 μM), FGF-8b (100 ng/mL), and CHIR 99021 (3 μM). Differentiated cells were stained with neural stem cell markers, SOX1 (R&D, AF3369), SOX2 (Stemgent, #09-0024) and NESTIN (Millipore, #mab5326).

Directed differentiation of hESCs to cardiomyocytes was achieved by temporal modulation of glycogen synthase kinase 3 (GSK3) inhibition followed by Wnt inhibition as previously described [20]. Briefly, blood reprogrammed lines were seeded at 2.5 × 10^5^ cells per well of 24-well coated plates and maintained with mTeSR1 for 3 days. On Day 0 of differentiation, medium was replaced with RPMI plus B-27 without insulin (Life Technologies, #0050129SA) plus GSK3 inhibitor CHIR99021 (Stemgent, 12 μM) for 24 h. On Day 1 of differentiation, CHIR99021 was removed and replaced with RPMI plus B-27 minus insulin alone until Day 3. On Day 3 of differentiation, medium was replaced with RPMI plus B-27 without insulin plus Wnt inhibitor 2 (IWP2, Stemgent, 5 μM) for 48 h until Day 5. On Day 5 of differentiation, IWP2 was removed and replaced with RPMI plus B-27 without insulin until Day 7. On Day 7 of differentiation, medium was replaced with RPMI plus B-27 and subsequently maintained by exchanging RPMI B-27 every 3 days. Differentiated cells were stained with cardiomyocyte markers, NKX2.5 (Santa Cruz, #sc-14033), Troponin/cTnT (Bioss, #bs-2804), and ACTIN (Sigma-Aldrich, #A7811).

Directed human hepatocyte-like cell differentiation were first induced to definitive endoderm (DE) by treating with STEMdiff Definitive Endoderm kit (Stem Cells Technologies, #05110) for 5 days, followed by the treatment of 50 ng/ml Activin A (R&D Systems) for 3 days. To induce hepatoblast formation from DE, the cells were then cultured with serum-free differentiation medium supplemented with 10 ng/ml FGF10 (R&D Systems), 10 ng/ml bone morphogenetic protein 4 (BMP4, R&D Systems) for 4 days. During the hepatocyte commitment and maturation stage, cells were cultured with HCM hepatocyte culture medium (Lonza) containing 50 ng/ml HGF (R&D Systems) and 20 ng/mL oncostatin M (OSM, R&D Systems) for 7 days. Differentiated cells were stained with hepatocyte cell markers ALB (R&D Systems, #MAB1455), AFP (Abcam, #ab3980). Albumin (ALB) secretion levels were determined by Albumin Human ELISA Kit (Abcam, #ab108788). The ability of the hepatic-like cells to store glycogen was calculated by Periodic acid-Schiff (PAS) staining (Sigma, #395B).

### Albumin ELISA Assay

Albumin concentrations in supernatants were detected by ELISA assay as Abcam Human Albumin ELISA Kit (Abcam, #ab108788) according to the manufacturer’s instructions. Briefly, conditioned media from undifferentiated cells and blood-iPSC derived hepatocyte-like cells were collected at Day 20. One hundred microliters of each standard, control, and samples were loaded to each well and incubated for 1 h, followed by five washes. The plate was incubated with biotinylated human albumin detection antibody for 30 min, washed five times, and incubated with SP conjugate for another 30 min, washed five times, immersed in chromogen substrate for 20 min. After adding stop solution, the plate was read at 450 nm using an absorbance reader (BioTek ELx800). The concentration of human albumin was calculated according to a four-parameter logistic equation.

### Periodic Acid-Schiff (PAS) Staining

PSA staining was performed using Periodic acid-Schiff Kit (Sigma, #395B) according to manufacturer’s instructions with minor adaptations. Briefly, cells were fixed in 4 % formaldehyde for 1–2 min, washed with PBS, stained for 5 min with 1 % periodic acid, and washed with distilled water prior to incubation with Schiff’s reagent for 15 min. After washing three times with water, cells were counterstained for 90 s with hematoxylin solution, and washed again three times with water prior to microscopic examination and imaging.

### Karyotyping and G-banding

GTG-banding chromosome analysis was carried out in Cell Line Genetics Laboratories. Standard DNA spectral karyotyping procedures were followed and a HiSKY Complete Cytogenetic System was used (Applied Spectral Imaging, Vista, CA). Data were interpreted by a certified cytogenetic technologist.

## Results

### Blood Storage and PBMC Preparation

Given the frequent instances in which blood might not be processed immediately, a question has arisen as to how best to store a good quality blood sample. Three methods have been tested to store four blood samples by evaluating the cell viability following cryopreservation and thawing. Since cell viability counting is a highly subjective assay, with significant variation in results among different personnel when analyzing the same cell sample, a robotic system Countess (Life Technologies) was used to assess viability using trypan blue as an assay reagent. Method One is termed as SAF: blood samples were mixed with one volume of SAF, and were centrifuged to get the cell pellets. The cell pellets were resuspended with SAF and cryopreserved in a liquid nitrogen tank; Method Two is termed as DMSO: blood cells were cryopreserved after mixing with 10 % cryoprotectant DMSO; Method Three is termed as VCPT: PBMC were isolated using Vacutainer Cell Processing Tubes (VCPT), and cryopreserved with SAF. We observed 66.7–78.3 % cell viability in blood cells processed by method one, 41–63.9 % cell viability in cells prepared by method two, and 87.5–96 % cell viability in isolated PBMC samples that were isolated by method three (Supplemental Figure S1B). Thus, the results obtained indicate that the VCPT method would give us the best cell viability but it requires additional time and an aseptic environment for cell preparation. The second method leads to death of about 50 % of the cells, however it is the fastest and most convenient way to store blood samples if cell processing is not allowed before cell cryopreservation. The first method could also be a fast and convenient way to store viable whole blood samples with limited processing.

As individuals have varying number of white blood cells, we sought the volume range of frozen peripheral blood needed for reprogramming. We evaluated the efficiency of PBMC isolation using vacutainer cell preparation tubes (BD), and used the value of PBMC yield and cell viability to determine the volume range of cryopreserved peripheral blood needed for iPSC generation. PBMC were isolated from eight donors with yields varying at 0.43–4.5 × 10^6^/mL with 88–98 % cell viability (Supplemental Figure S1A).

### High Efficiency of Human PBMC Reprogramming Using a Low Attachment Plate

We used Sendai viral vector to reprogram blood cells because it is a negative-sense RNA virus with no potential for gene integration with concomitant by high transduction efficiency. Current protocols for reprogramming peripheral blood cells are based on CytoTune-iPS 2.0 Sendai Reprogramming Kit (Life technologies) with modifications.

**Fig. 2:**
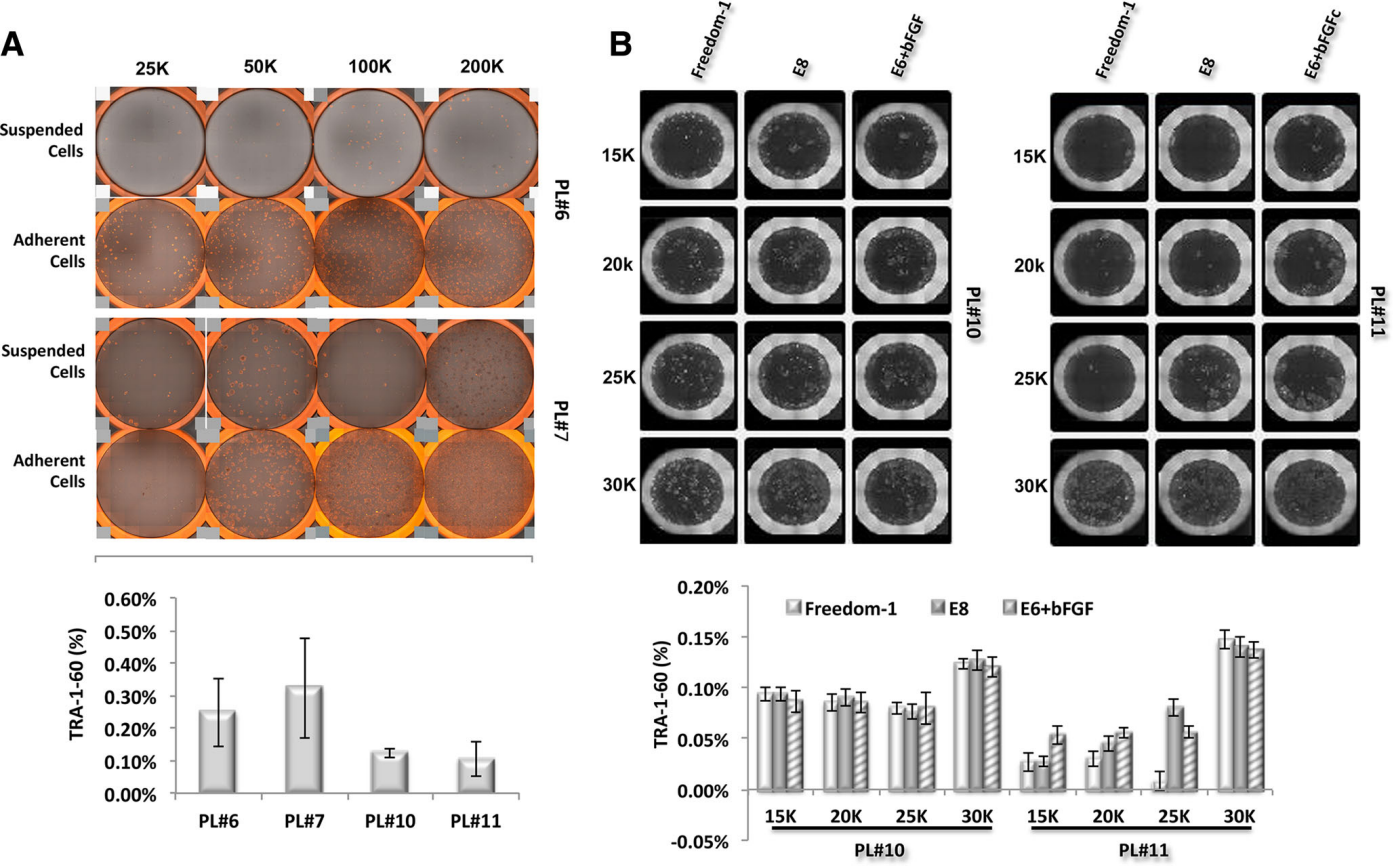
Seeding density and attachment effect on peripheral blood reprogramming. **a**, *upper panel*: representative live whole-well TRA-1-60 images from wells of 24-well plates containing different seeding number. After 4 days cell expansion, the cell in suspension were collected for reprogramming induction, and named as Suspended Cells; the cell attached to the plate bottom were dissociated with 0.5 mM EDTA and transfected with reprogramming factors and termed as Adherent Cells. Images were recorded by Celigo software 10 days post reprogramming factors transduction. *Lower panel*: reprogramming efficiency for each peripheral blood samples. Efficiency was calculated based on total TRA-1-60 colony count from both suspended cells and adherent cells for each seeding density. **b**, *upper panel*: representative live whole-well TRA-1-60 images from wells of 96-well plates containing different seeding number. Three kinds of hESC medium were tested in the reprogramming assay (Freedome-1, E8, and E6+bFGF), images were recorded by Celigo software 10 days post reprogramming factor transduction. *Lower panel*: reprogramming efficiency for each condition was calculated based on the total TRA-1-60 colony count

In our initial experiment, PBMCs were seeded on standard 24-well plates. The cells were cultured in the presence of IL3, IL6, SCF and FLT3 for 4 days to expand the hematopoietic progenitors, and transfected with Sendai virus encoding *Oct4, Sox2, Klf4*, and *cMyc*. After 2 days of recovery, the transduced blood cells were transferred onto Geltrex (Life Technologies) coated plates and cultured without cytokines for another 4 days. At Day 7, the cells were incubated with customized human ESC medium Freedom-1 (Life Technologies). The first iPSC colonies appeared approximately 1 week after transfection (Day 7, Fig. 1b). To determine the appropriate cell number needed for reprogramming, we set up a cell seeding density experiment in standard 24-well plates (25 to 200 k per well of 24-well plates). We found although most of the cells stayed in suspension, some cells spontaneously attached to the culture surface during the 4-day cell expansion (for instance, for PL#7, we observed more cells attached to the culture surface from 50 k seeding well than from 25 or 100 k seeding wells). Both the attached and suspended cells can be reprogrammed as demonstrated by the appearance of TRA-1-60+ colonies, though the reprogramming efficiency varies from sample to sample (0.15–0.32 %, Fig. 2a). We observed that initial seeding at 25–50 k per well of 24-well plates result in a uniform distribution of TRA-1-60 positive colonies, while there are too many colonies to distinguish if more than 100 k cells were seeded (Fig. 2a).

In order to reduce the variation introduced by cell attachment and cell loss during cell dissociation, we next studied the applicability of mononuclear cell expansion in 96-well low attachment plates. The results (Fig. 2b) indicate that culturing PBMCs in low attachment did not compromise the reprogramming efficiency (representative results of donor line PL#10 and PL#11).

In addition to the application of Freedom-1 medium during iPSC reprogramming, we also examined the effect of the other GMP-grade xeno-free media, Essential E8 (Life Technologies) and E6 (Life Technologies) supplemented with 10 ng/ml bFGF (as the presence of TGFβ in E8 medium may inhibit early stage cellular reprogramming). As shown in Fig. 2b, we did not observe notable differences in reprogramming efficiency in the three medium treatments. The established iPSC lines used in the following characterization were maintained with Freedom-1 medium.

### Characterization of Expanded PBMC Culture

**Fig. 3:**
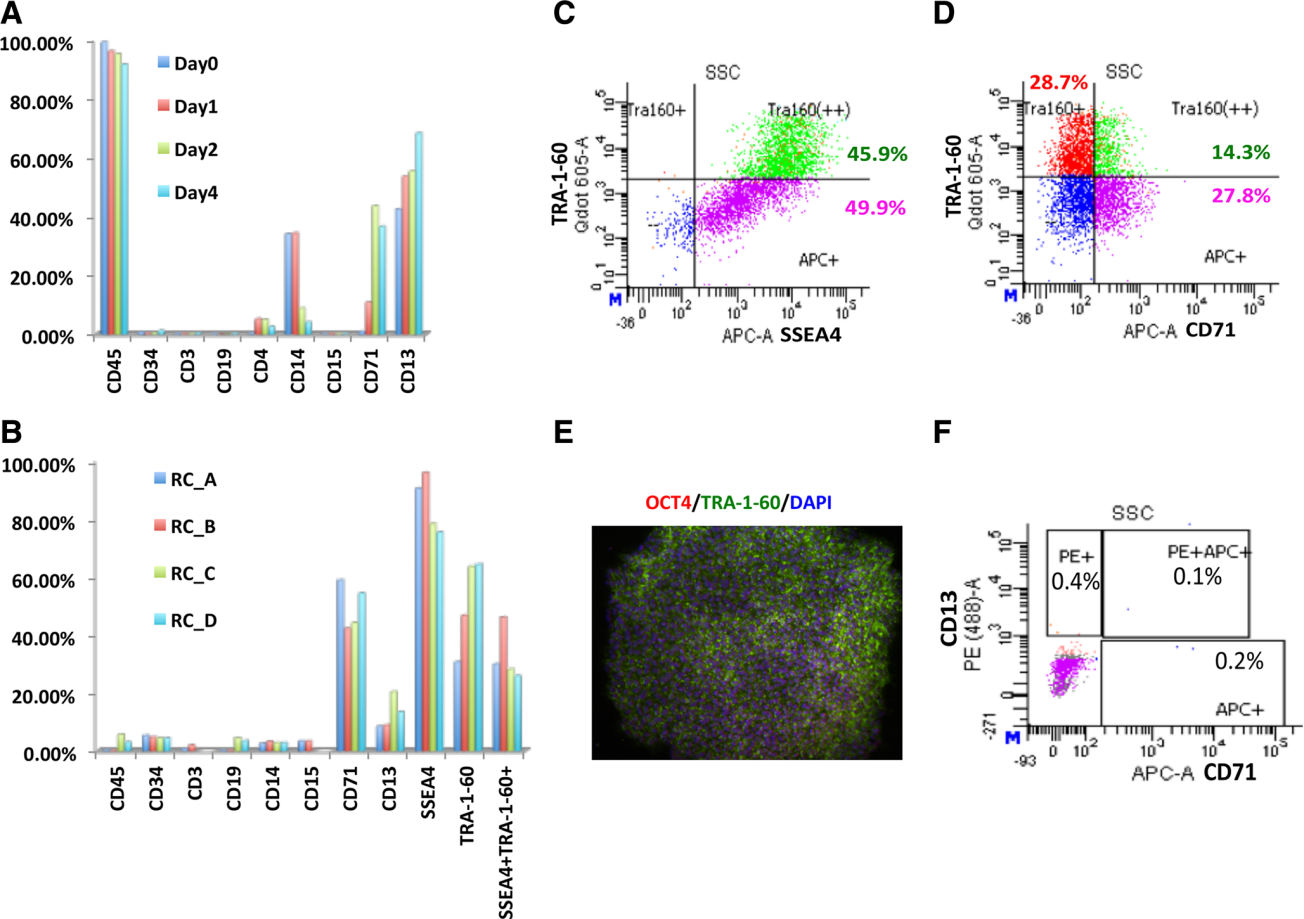
Cell fate characterization. **a**, Expression of blood sublineage cell surface markers in PBMC during 4-day cell expansion. **b**, Expression of blood sublineages and common pluripotency markers in four independent reprogramming cultures RC_A, RC_B, RC_C, RC_D, collected on day 14 after reprogramming induction. **c**, Flow cytometry analysis of SSEA4 and TRA-1-60 expression on representative reprogramming culture RC_B. **d**, Flow cytometry analysis of CD71 and TRA-1-60 expression on representative reprogramming culture RC_B. **e**, Purified iPSCs express pluripotency markers OCT4 and TRA-1-60. DAPI services as a control, **f**, Flow cytometry analysis of CD71 and CD13 expression on purified iPSCs

As mononuclear cells are a heterogeneous group of nucleated cells, incubation of mononuclear cells with a combination of cytokines could selectively expand one or several types of blood cells. We next asked what cell type(s) are reprogrammed using this protocol by characterizing the cell fates during cell expansion and reprogramming. During the 4-day PBMC expansion period, a portion of mononuclear cells were collected daily for characterization using flow cytometry. At the end of the 4-day cell expansion, an 80-200–fold expansion of CD71+ erythroblast, a ~1.5–fold expansion of CD13+ cell, a slight increase of CD34+ progenitors, and a decrease in the percentage of CD14 (~12 fold decrease) were observed. While >95 % of the cells manifested a hematopoietic cells characteristic (CD45+), this culture condition did not expand mature T-lymphocytes (CD3+) and B-lymphocytes (CD19+), as indicated by decreased and low percentage of cells present in the expanded cell culture (Fig. 3a, 0.21–0.40 % CD3+ and 0.03–0.64 % CD19+). These observations indicated that CD13+ myeloid progenitors and/or CD71+ erythroblast cells are dominant after 4 days of cell expansion.

### Surface Marker Profile of PBMC-Derived Cell Culture Undergoing Reprogramming

We observed that iPSC colonies appeared 7–10 days after transfection identified by typical human ESC morphology and expression of pluripotent cell surface marker TRA-1-60 (Fig. 1b). Commonly used hiPSC purification strategies include manual colony picking, FACS, magnetic bead separation on TRA-1-60 or complement depletion. Among them, complement depletion offers the advantage of being able to adapt to automation platforms for large-scale production [22]. Therefore, we next sought to find the surface markers that allow the purification of iPSC from the refractory bulk populations by depleting the un-reprogrammed cells. Four independent reprogramming cultures (RC_A, RC_B, RC_C, RC_D) were picked randomly on day 14 after induction of reprogramming. The bulk populations of each reprogramming culture were dissociated and subjected to FACS analysis using blood sublineages and common pluripotency markers.

We found significant molecular changes that indicate the commitment to the iPSC cell fate: the appearance of pluripotent cell surface markers SSEA4+ (75.1–95.8 %) and TRA-1-60+ (45.5–63.6 %) was paralleled by a significant decrease in the expression of the pan-leukocyte receptor CD45+ (before reprogramming 89.4–92.9 % versus after reprogramming 0.10–5.3 %), and a decrease in the expression of CD13 (before reprogramming 58.3–69.1 % versus after reprogramming 9.6–20.3 %) (Fig. 3a and b). We also observed a certain degree of heterogeneity in the reprogramming populations: approximately half of the SSEA4+ cells have expression of TRA-1-60, suggesting they are at a more mature iPSC stage as early as at 14 days post induction of reprogramming (Fig. 3c). The finding that these SSEA4+TRA-1-60+ cells also express CD71 and/or CD13 (Supplemental Figure 2) indicates their CD71+ or CD13+ origins. A portion of CD71+ cells becomes double positive for TRA-1-60 (Fig. 3d), further suggesting that cells are CD71+ origin and in the transition stage of converting from CD71+ to iPSCs.

We next purified iPSC by depleting un-reprogrammed CD71+ and CD13+ cells using anti-CD71 magnetic beads and anti-CD13-Biotin microbeads (Miltenyi). The purified cells were seeded on Geltrex-coated plates and subsequently cultured for 6 days to re-establish iPSC colonies. Immunohistochemistry results showed that these cells express pluripotent markers OCT4 and TRA-1-60 (Fig. 3e) suggesting their pluripotency, and FACS analysis data demonstrate that the cells were completely eliminated the CD13 and CD71 population in the purified iPSCs (Fig. 3f).

### Blood Derived iPSCs are Free from Gene Rearrangements and Transgenes

**Fig. 4:**
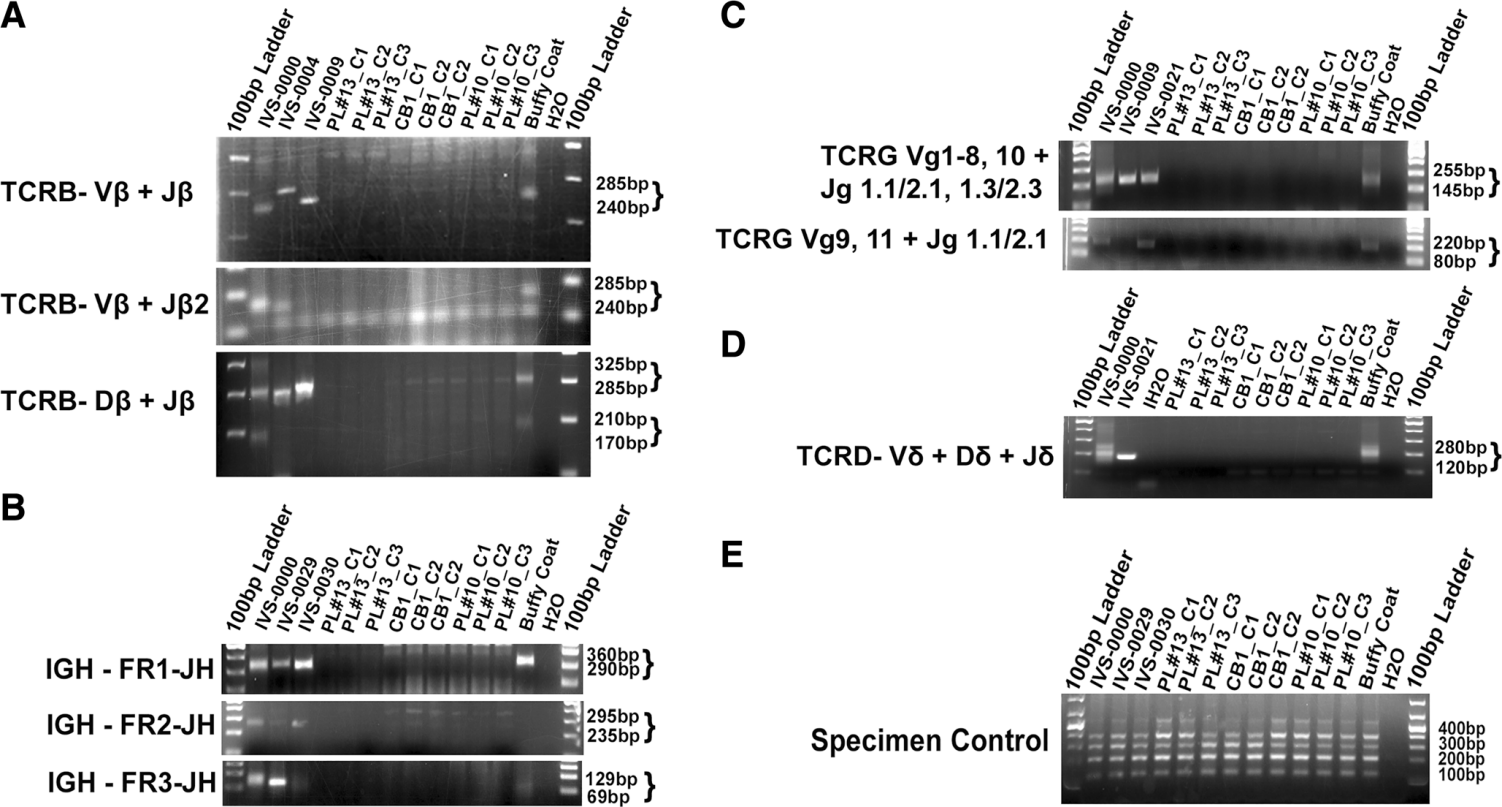
TCR and IGH rearrangement analysis in PBMC and CB iPSCs. **a**, PCR analysis of TCRB rearrangements. **b**, PCR analysis of IGH rearrangements. **c**, PCR analysis of TCRG rearrangements. **d**, PCR analysis of TCRD rearrangements. **e**, Specimen control. IVS-0000: polyclonal control DNA; IVS-0004, IVS-0021, IVS-0030: clonal control DNA; H2O: no template control; Buffy coat: genomic DNA from buffy coat PL#10

Since iPSCs carrying pre-existing gene rearrangement may have uncertain functional consequences [12, 23], we used PCR to detect clonal IGH and TCR gene rearrangements in iPSCs to determine if they were derived from B- or T- lymphocytes. As shown in Fig. 4, whereas the clonal control and parental whole blood cells showed bands reflecting the presence of gene rearrangements, all tested iPSC lines did not show bands in the valid size range for TCRB_Vβ + Jβ, Vβ + Jβ2, Dβ + Jβ (Fig. 4a), TCRG (Fig. 4c), TCRD (Fig. 4d), and IGH gene rearrangements (Fig. 4b), indicating they were derived from non-B, non-T cells. This could be explained by the selective cell growth of CD13+ and CD71+ cells with the cell expansion cocktail (Fig. 3a).

**Fig. 5:**
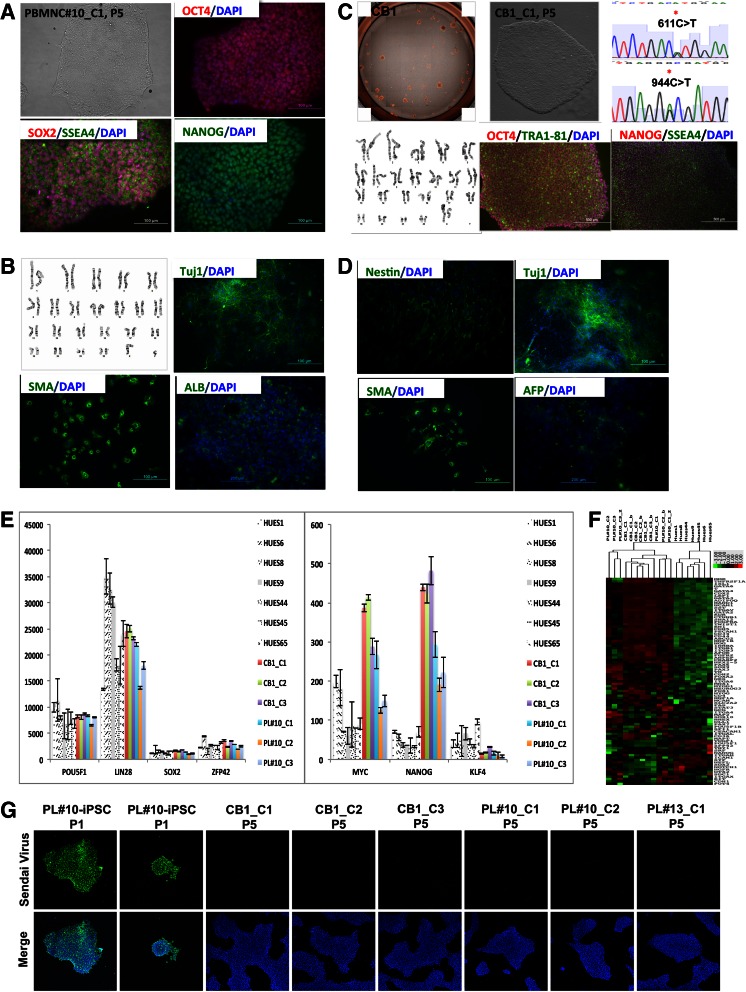
Characterization of iPSCs derived from PBMC and cord blood. **A** subset of iPSC clones was characterized. The experiments demonstrated in the figure provide representative examples of the results observed for iPSC clone 1, 2, 3 derived from peripheral blood donor PL#10, and iPSC clone 1, 2, 3 derived from cord donor CB1. **a**, PBMC derived-iPSCs show typical human ESC morphology express pluripotent markers OCT4, SOX2, SSEA4, NANOG detected by immunochemistry. **b**, Representative cytogenetic analysis on G-banded metaphase cells from iPSC clone 1 exhibiting a normal karyotype. iPSCs can form EB, spontaneously differentiate towards ectoderm (Tuj1), mesoderm (SMA), and endoderm (SMA), and endoderm (ALB). **c**, Approximately 3000 cord blood cells were efficiency reprogrammed. Picked colony shows typical human ESC morphology, express plurinent cell surface marker TRA-1-60, patient disease ELK3 gene mutations conforming the sample identity. **d**, Cord blood derived iPSCs can form EB efficiently, spontaneous differentiation results show their differentiation potential towards ectoderm (NESTIN, Tuj1), mesoderm (SMA), and endoderm (AFP). **e**, Pluripotent gene expression determined by quantifying RNA transcripts counts using nCounter Analysis System, *error bars* represent standard derivation of the mean (*n* = 2). **f**, Hierarchical Cluster of EBs. **g**, Detection of sendal viral vector by immunohistochemistry

In addition, the generation of iPSC free of integrated reprogramming factor genes is essential to reduce differentiation biases and artificial phenotype [24]. Immunochemistry staining with anti-Sendai antibody was negative for Sendai virus vector in all test colonies that were obtained at passage 5 (Fig. 5g), confirmed that Sendai virus vector were diluted out during the proliferation, and no transgene were carried in the iPSCs.

### PBMC-Derived iPSC Characterization

iPSC colonies were expanded into stable iPSC lines that displayed the morphology characteristic of human ESCs, stained positive for the pluripotency markers OCT4, SOX2, SSEA4 and NANOG (Fig. 5a), and had a normal karyotype (Fig. 5b). Nanostring Pluri25 analyses of pluripotent genes *Pou5f, Zfp42*, *Sox2*, *Klf4*, *Nanog*, *Lin28*, and *Myc* confirmed pluripotency (Fig. 5e). To assess the in vitro differentiation capacity of the iPSC lines, the cells were differentiated into EBs. Immunohistochemistry staining for spontaneous differentiation revealed that protein for all three germ layers including ectoderm (Tuj1), mesoderm (SMA) and endoderm (ALB) were present indicative of pluripotent status (Fig. 5b). Scorecard assay of ectodermal, mesodermal, and endodermal markers demonstrated that all three germ layer markers were expressed at a level similar to the reference human ESC lines (Fig. 5f).

### High Reprogramming Efficiency for Umbilical Cord Blood (UCB)

The same strategy was applied to reprogram mononuclear cells of UCB from a child enrolled in the Duke Task Force for Neonatal Genomics (TFNG), a whole exome sequencing and functional testing program aiming to accelerate gene discovery and diagnosis in young children with suspected genetic disease. Due to the limitation of valuable cell resource, we collected 3000 cells from a thawed segment of a cyropreserved cord blood unit, and applied MOI of 15 to induce reprogramming. As shown in the Fig. 5c, we obtained approximately 52 clones, with a reprogramming efficiency at 1.73 %. We manually picked six colonies to establish iPSC lines. All iPSC lines carry the causal gene mutations in *ELK3* (Fig. 5c) indicating they were derived from UCB cells harboring the mutations. All iPSC lines displayed the typical ESC morphology, free of gene rearrangements (Fig. 4). Three derived iPSC lines maintained a normal karyotype, and were free of Sendai Vectors (Fig. 5g). All iPSC lines could differentiate towards three germ layers (Fig. 5d), displaying similar gene expression patterns as hESC reference lines in both Nanostring Pluri25 and 3GL scorecard assays (Fig. 5e and f).

### Directed Differentiation of iPSCs Towards Functional Cells from Three Germ Layers

Next, we evaluated the differentiation potential of peripheral blood-derived iPSC (PBMC-iPSC) and cord blood-derived iPSC (CB-iPSC) into neural stem cells (NSCs), cardiomyocytes, and into hepatocyte-like cells that are used commonly for toxicity testing and predicting drug metabolism.

**Fig. 6:**
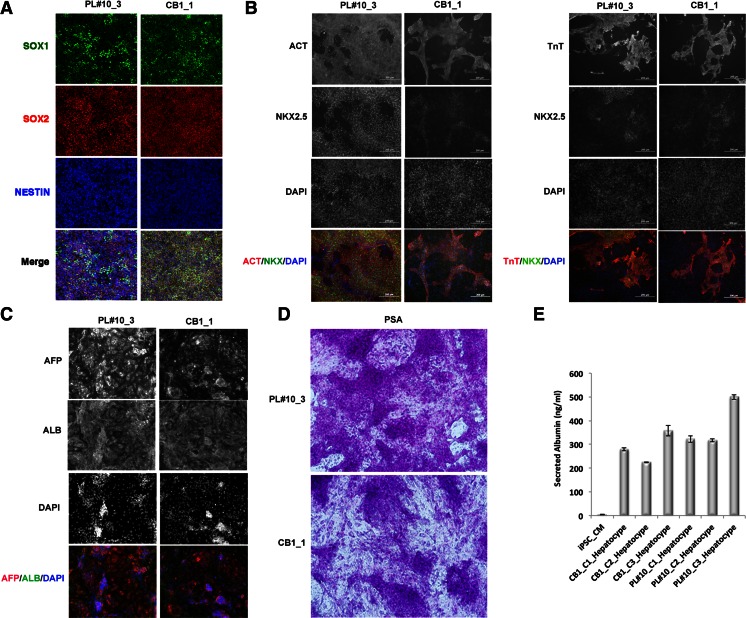
Directed differentiation of blood cell-derived iPSCs. Representative examples of the results observed for iPSC clone 3 derived from peripheral blood donor PL#10, and iPSC clone 1 derived from cord blood donor CB1. **a**, NSC differentiated from iPSCs. Cells stained with neural stem cell specific markers SOX1, SOX2 and NESTIN. **b**, Cardiomyocyte differentiated from iPSCs, cell stained with markers α-Actinin (ACT). NKX2.5, Troponin T (TnT), and DAPI. **c**, Hepatocyte-like cells differentiated from iPSC. Cells stained with α**-**fetoprotein (AFP), albumin (ALB), and DAPI. **d**, Periodic acid-Schiff (PAS) staining. *Purple color* in cells indicates glycogen accumulation. **e**, Albumin secretion by CB_iPSC derived hepato-cyte-like cells was assayed by ELISA. The differentiation medium was changed to fresh medium 24 h before the assay. The concentration of the ALB secreted from 30 k iPSC_derived hepatocyte-like cells in 200 ul of media was measured on differentiation Day 16. *Error bars* represent the standard deviation of the mean (*N* = 2)

We applied a multistage differentiation protocol developed previously to promote the conversion of human iPSCs into NSCs [18, 19] via inhibition of TGFβ and BMP signaling in a defined cell monolayer system. The treatments resulted in the emergence of typical NSCs that express marker proteins such as SOX1, NESTIN, and SOX2 at a similar efficiency across the iPSC lines (95 ± 5.6 %) (Fig. 6a).

In vitro cardiomyocyte differentiation of iPSCs proceeded through modulating Wnt/b-catenin signaling [20]. We found all PBMC-iPSC and CB-iPSC lines followed a similar pattern of cardiomyocyte differentiation, could form spontaneously contracting areas, and the contracting myocytes were stained positively with anti-α-Actin and anti-cTnT, although variation in differentiation efficiency was observed among different iPSC lines. Testing of three PBMC-iPSC clones revealed similar differentiation kinetics and marker expression profiles (Fig. 6b).

In vitro hepatocyte-like cell differentiation of PBMC-iPSC and CB-iPSC was performed as described previously [21]. The PBMC-iPSC and CB-iPSC lines were differentiated using a step-wise, efficient approach into hepatocyte-like cells that show cobblestone morphology, bi-nucleation, express hepatocyte specific genes such as AFP, ALB (Fig. 6c), and exhibit glycogen storage (measured by PSA staining) as well as phenotypic characteristic of hepatocytes like albumin secretion (Fig. 6d and e).

## Discussion

Production of blood cell derived iPSC is currently a costly and tedious endeavor, limited by low efficiency and slow kinetics. Here we show blood reprogramming can be achieved within 2 weeks from cell collection to iPSC purification and can be scaled up to 96-well plates format, thus simplifying and streamlining the manufacturing process, and enabling the adaption to medium/high-throughput hiPSC production.

We demonstrate that blood cells from a small tube segment attached to a cryopreserved cord blood unit are sufficient to generate iPSC lines without compromising their ultimate therapeutic use. This is an important advance to permit access to numerous frozen samples already stored at blood banks since the samples are often of restricted use for research due to limited cell numbers for therapeutic purposes. The minimal volume of blood materials required for reprogramming varies for each individual as the mononuclear cell density varies. When fresh peripheral blood is obtained from normal donors, the yields are in the range of ~0.5–4 × 10^6^ mononuclear cells/mL with a viability >95 % (Supplemental Figure S1B). Mononuclear cells from frozen peripheral blood would have a lower yield due to cell damage during cryopreservation. In this study, we tested three cryopreservation methods, and found even freezing cells using 10 % DMSO mixed with whole peripheral blood will have ~50 % cell viability on recovery (Supplemental Figure S1A), thus offering a not so optimistic but very convenient method to collect samples especially where no applicable sterile environment exists for mononuclear processing. We demonstrated that ~30,000 mononuclear cells can be efficiently reprogrammed using Sendai viral vectors. Assuming that yields of mononuclear cells from peripheral blood are 1 × 10^6^/mL and 50 % of cells would be lost during cell cryopreservation, approximately 60 μl of frozen peripheral blood will be needed for iPSC generation, making the iPSC technology more broadly applicable to vulnerable individuals, such as pediatric patients with severe medical challenges and unique HLA sample segments without disturbing the blood units. For those valuable samples with limited available cells, reprogramming could be performed using higher MOI Sendai viral vector to increase the efficiency.

**Fig. 7:**
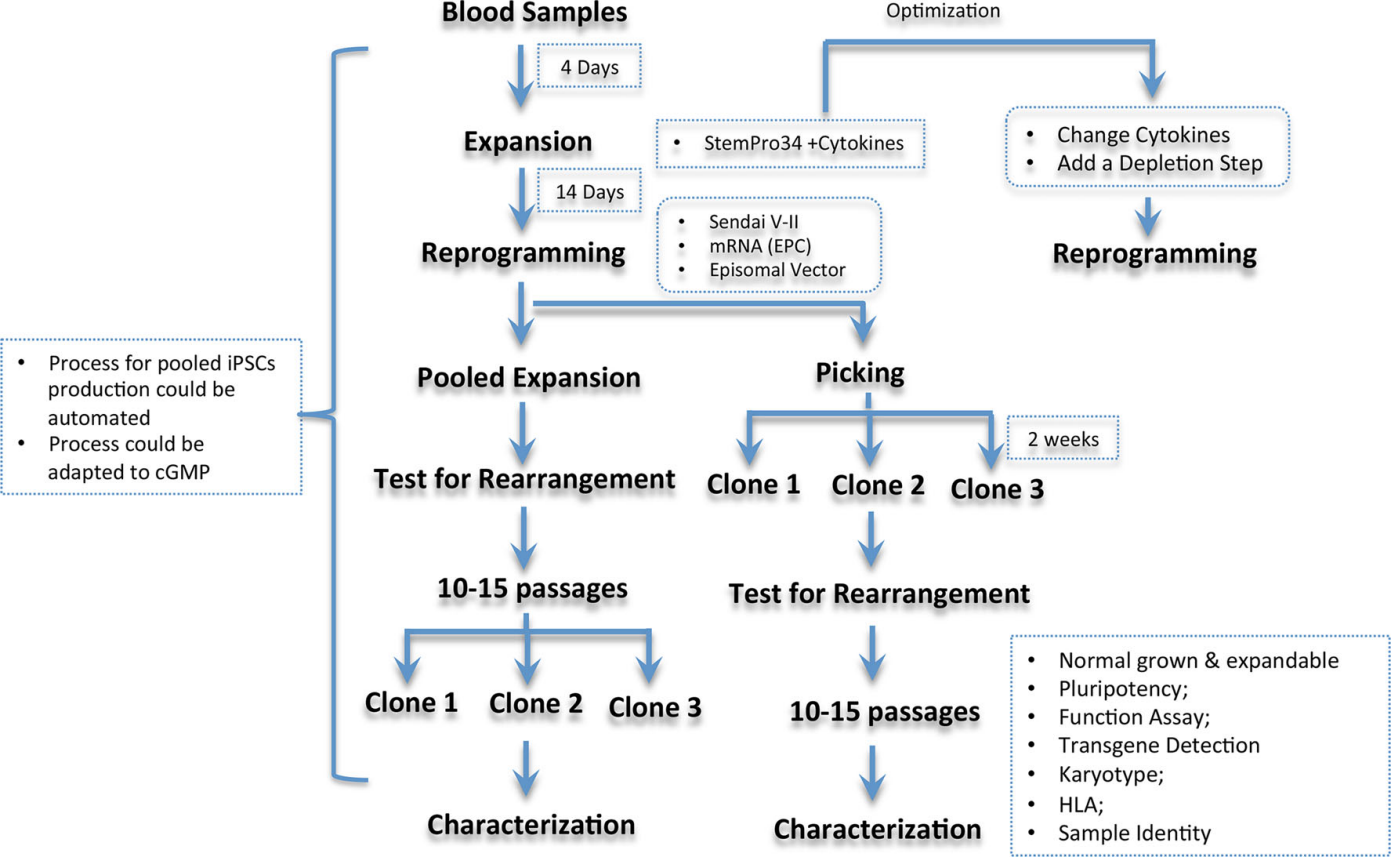
Flow chart of blood derived iPSC production

Generation of iPSC colonies that carry gene rearrangements could be avoided by selectively expanding and using non-T, non-B cells for reprogramming. To the best of our knowledge, the present study is the first to examine the cell expression pattern of CD13 and CD71 during cell expansion and after cellular reprogramming. Our protocol is shown to be robust and much faster than a previous report that showed a 14-days cell expansion was needed to expand erythroblast for gene-rearrangement-free iPSC generation [25]. We were able to selectively expand the CD13+ and CD71+ progenitors using a brief 4-day cell expansion before reprogramming, providing cell materials to generate iPSC lines without gene rearrangements (Fig. 3). In this study, we applied a traditional PCR assay to detect the gene rearrangement, while in an automated cell line production scenario, a high-throughput screening method like Illumnia MiSeq could be used to verify gene rearrangement status. The medium recipe could be optimized further if a unique cell population is desired by adjusting the cytokines or depleting any unwanted cell population (Fig. 7).

Once the blood reprograms into iPSC cells, colonies could be manually picked and expanded for further characterization. Another option is to purify the iPSC via positive selection (i.e., using anti-TRA-1-60 that specifically binds to iPSC) or negative depletion. Using FACS analysis, we found those un-reprogrammed cells in the bulk population are mainly CD13+ and CD71+ cells, thus depletion of CD13+ and CD71+ cells may be used to achieve a purer iPSC population, offering an adaptable method to automated cell production systems. The disadvantage of this strategy is that the iPSCs are from a bulk population and not a single colony. However, colonies could be picked manually at any stage after bulk purification if desired.

In summary, our study allows reprogramming of the most readily available blood cells and provides a protocol to access samples stored at blood banks, enabling the blood collection from pediatric and other populations that cannot easily provide much peripheral blood or a skin biopsy. The iPSC derived from such blood samples could provide unlimited cell sources to retrospectively screen for genetic factors, to study molecular mechanisms underlying blood disorders or other diseases, to derive the other functional differentiated cells for drug screening and disease modeling, and could even potentially used for cell therapy.

## Electronic supplementary material

Below is the link to the electronic supplementary material.

Supplemental Figure S1 (GIF 63 kb)

High Resolution (TIFF 15611 kb)

Supplemental Figure 2 (GIF 87 kb)

High Resolution (TIFF 14877 kb)
