# Supplementary figures and images for: Rapid and Efficient Generation of Transgene-Free iPSC from a Small Volume of Cryopreserved Blood
Source: Stem Cell Rev. 2015 May 8;11(4):652–65. doi: 10.1007/s12015-015-9586-8 (PMC4493720; doi:10.1007/s12015-015-9586-8)

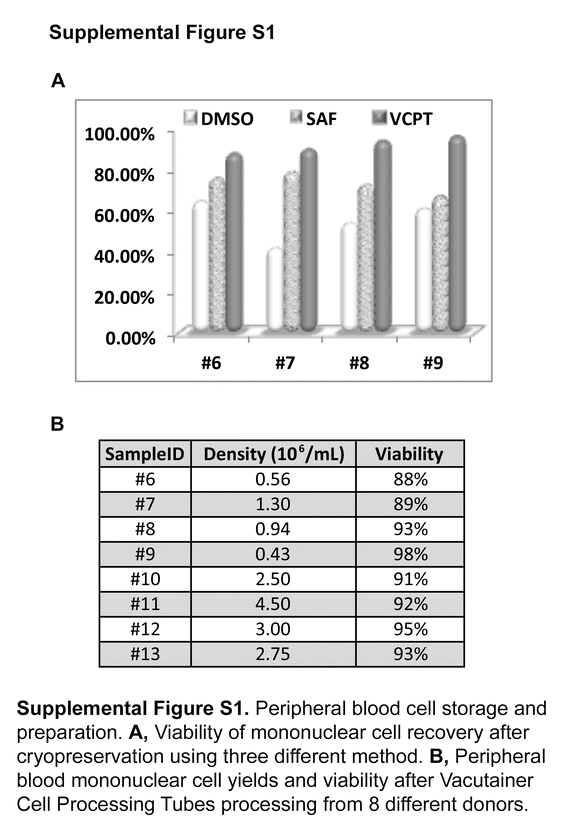

Supplement: Supplementary file 1 — (GIF 63 kb) [file 12015_2015_9586_Fig8_ESM.gif]

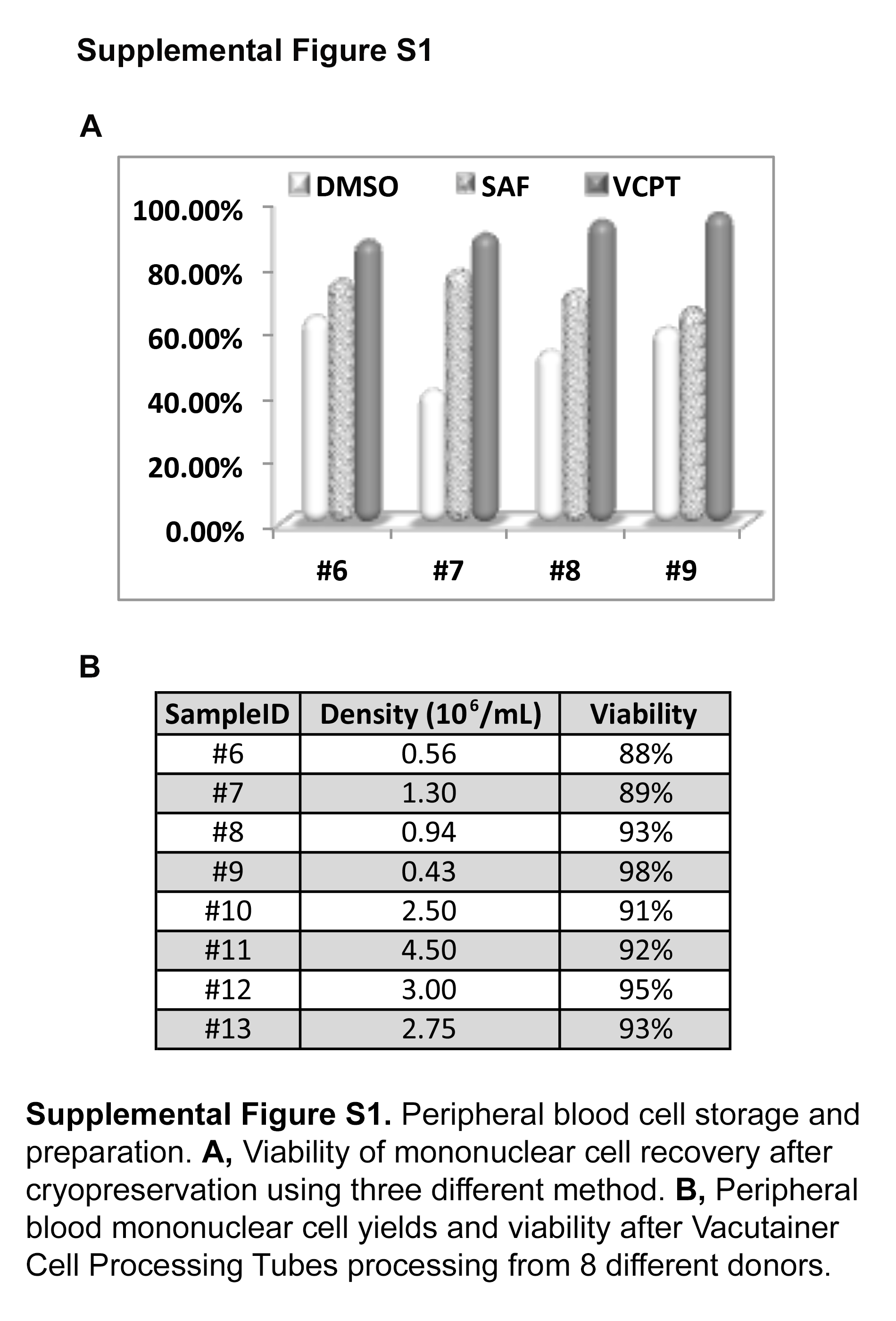

Supplement: Supplementary file 2 — High Resolution (TIFF 15611 kb) [file 12015_2015_9586_MOESM1_ESM.tif]

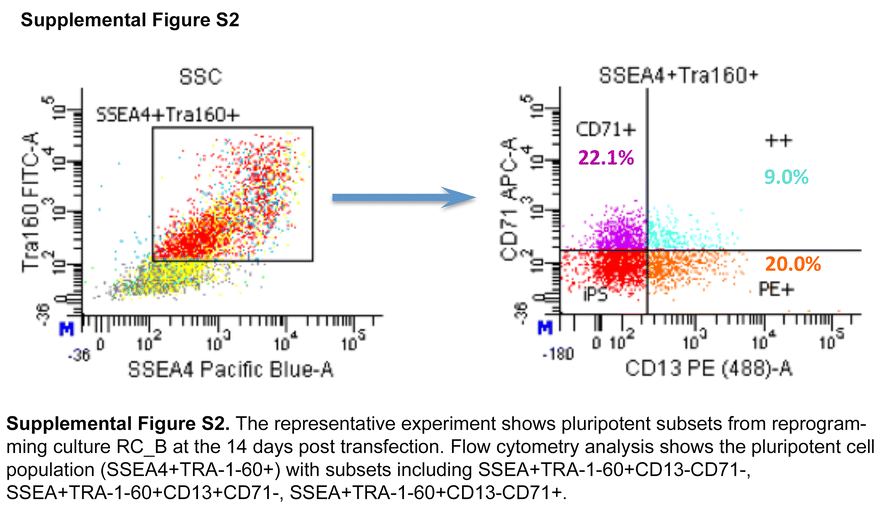

Supplement: Supplementary file 3 — (GIF 87 kb) [file 12015_2015_9586_Fig9_ESM.gif]

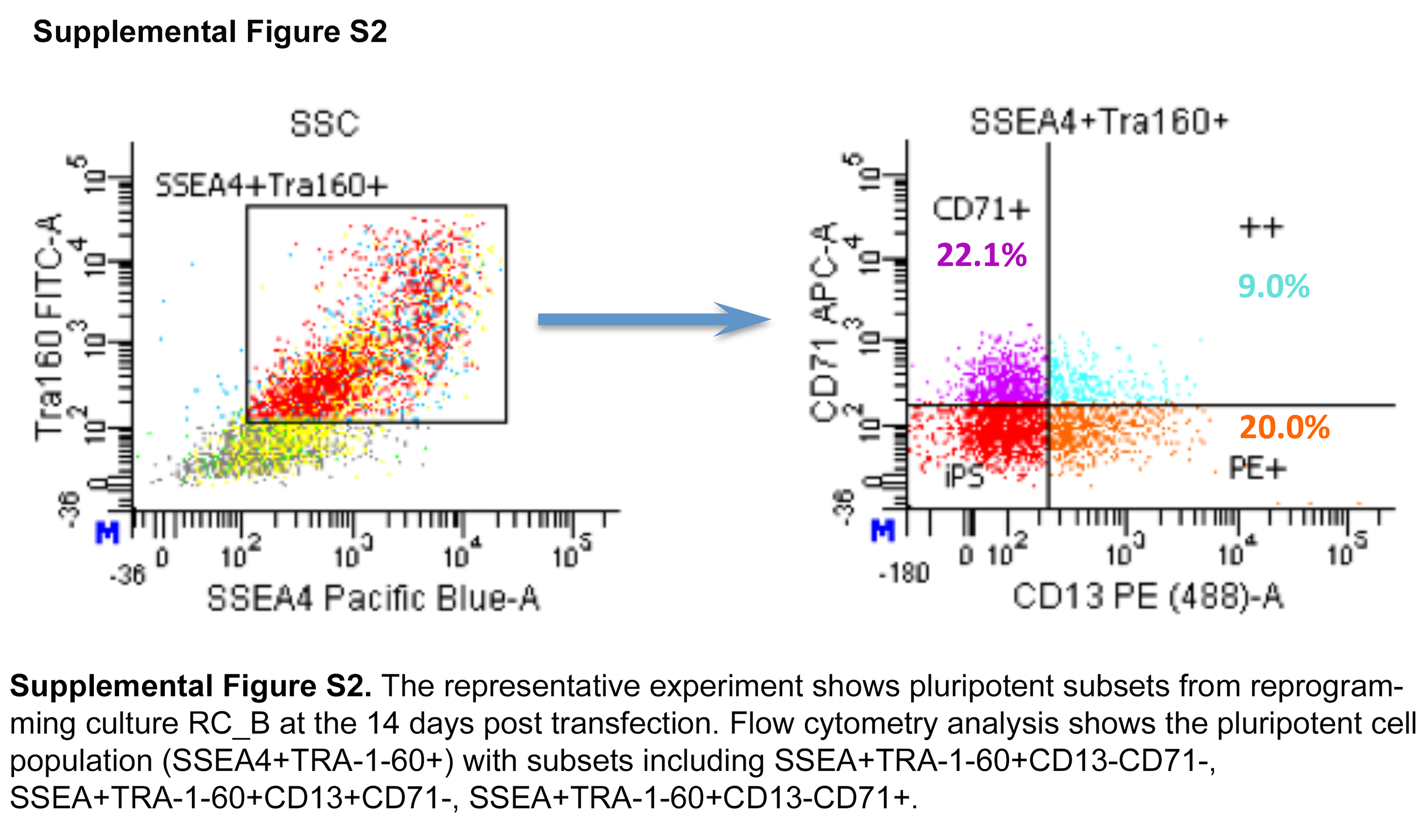

Supplement: Supplementary file 4 — High Resolution (TIFF 14877 kb) [file 12015_2015_9586_MOESM2_ESM.tif]
